# Towards a Sustainable Food Supply Chain: Digital Transformation via Blockchain, IoT and AI

**DOI:** 10.1002/fsn3.72422

**Published:** 2026-10-09

**Authors:** Zainab Jabbar, Atif Liaqat, Samreen Ahsan, Rabia Iqbal, Adnan Khaliq, Muhammad Farhan Jahangir Chughtai, Furukh Faiz, Muhammad Zahid Bashir, Tariq Mehmood, Edouard Bugingo, Muhammad Zubair Khalid

**Affiliations:** ^1^ Institute of Food Science and Technology Khwaja Fareed University of Engineering and Information Technology Rahim Yar Khan Pakistan; ^2^ Department of Agriculture and Food Technology Karakoram International University Gilgit Gilgit Pakistan; ^3^ Guangdong Provincial Key Laboratory of Aquatic Product Processing and Safety, College of Food Science and Technology Guangdong Ocean University Zhanjiang China; ^4^ Department of Food Science and Technology, College of Science and Technology University of Rwanda Kigali Rwanda; ^5^ Department of Food Science, Faculty of Life Sciences Government College University Faisalabad Punjab Pakistan

**Keywords:** artificial intelligence (AI), blockchain, digital transformation, internet of things (IoT), smart agriculture, supply chain management (SCM), sustainability

## Abstract

Supply chain management (SCM) plays a pivotal role in achieving competitive advantage, enhancing customer value, and ensuring efficient transformation of raw materials into finished food products. However, the increasing complexity and globalization of agri‐food supply chains (AFSCs) have intensified challenges related to food safety, waste management, traceability, and sustainability. In response, digital transformation driven by Industry 4.0 technologies, particularly Blockchain, Internet of Things (IoT), and Artificial Intelligence (AI), has emerged as a critical enabler of sustainable food systems. This review critically examines the role of these technologies in enhancing transparency, efficiency, and resilience across food supply chains. Blockchain ensures secure, immutable, and transparent data sharing, thereby improving traceability and trust among stakeholders. IoT facilitates real‐time monitoring of environmental and logistical parameters, reducing post‐harvest losses and ensuring product quality. AI‐driven analytics enable predictive decision‐making, demand forecasting, and resource optimization, contributing to reduced food waste and improved operational performance. Collectively, these technologies support precision agriculture, smart logistics, and data‐driven supply chain integration, thereby advancing sustainability goals. Despite these benefits, the adoption of digital technologies faces significant barriers, including high implementation costs, interoperability challenges, data privacy concerns, and limited technological infrastructure. This study highlights current advancements, identifies research gaps, and proposes future directions for scalable and secure digital integration to achieve sustainable and resilient food supply chains.

## Graphical abstract

Digital transformation in Agri‐Food Supply Chains (AFSCs) leverages Artificial Intelligence (AI), Blockchain, and Internet of Things (IoT) to enable real‐time monitoring, precision farming, smart agricultural logistics, and data‐driven decision‐making. Global trade frameworks, sustainable production paradigms, and environmental policies such as the European Green Deal and Farm‐to‐Table initiatives catalyze this digital shift to enhance farm‐to‐fork transparency and minimize food waste. Despite the benefits of enhanced traceability and operational efficiency, full‐scale adoption faces critical infrastructure and governance challenges, primarily concerning data privacy, cybersecurity, and technical interoperability across the supply chain network.

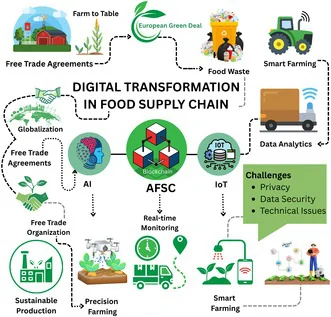

## 1 Introduction

A collection of procedures and sub‐processes that are used to convert raw material into finished goods, maximize customer value, and establish a sustainable competitive edge are known as supply chain management (SCM). A network of entities that are a part of the system from production to trade is another way to perceive it. Food waste, affecting one‐third of human consumption, leads to environmental, economic, and societal issues. It increases food insecurity, generates greenhouse gas emissions, and results in over $1 trillion annual economic losses (Santeramo and Lamonaca 2021). Sustainable food supply chains can benefit from integrating natural bioactive‐based strategies for mitigating risks associated with chemical and environmental contaminants (Bashir et al. 2024). In the agri‐food supply chain, there are serious issues of food loss and waste that can occur from a diversity of causes like inedibility, weight loss, pests, supply and demand imbalances. To reduce food insecurity, the United Nations Environment Programme (UNEP) emphasizes the significance of striking a balance between food loss and waste production and consumption. To solve these problems, the UN has created a model for a sustainable food system (Royer 2024). Food loss can happen on or off farms, and table waste can also lead to food waste because of carelessness and improper treatment (Bhat and Jõudu 2019). By the year 2050, Europe has been projected to be the first continent to reach the goal of being climate neutral; this is according to the European Green Deal of 2020. To make sure that none is left behind, to support the economy, to sustain the environment, as well as to enhance the health of the public and improve the quality of their life. This project offers a new approach to growth, which is sustainable and inclusive. The Farm to Fork approach, which addresses the challenges of sustainable food systems and strives to offer primary producers who are already struggling with income disparity a stable source of income, is at the center of the Green Deal (Sermuksnyte‐Alesiuniene et al. 2021; Bukhari et al. 2025). This is pivotal for the recovery and transformation to be successful. The food value chain has seen logistical problems due to the Covid‐19 epidemic. Businesses are under more and more pressure to produce value and provide it to customers in a cutthroat global environment. As a result, more effective logistics systems have been created to compete in terms of price, quality, and service. Technology advancements like blockchain and AI can improve supply chain efficiency (Arowosegbe et al. 2024). The demand for food is predicted to increase by 59% to 98% by 2050, necessitating the adoption of novel techniques and increased crop output by farmers (Lioutas and Charatsari 2020). Transparency and traceability have increased due to agricultural technology, such as automated irrigation systems and remote‐controlled harvesting. Stakeholders in the food supply chain must forge close ties between sustainable practices and the food value chain in light of consumers' growing interest in sustainability and compliance (Casino et al. 2021). Sustainability is transforming the global food supply chain, with technological tools like artificial intelligence (AI), machine learning (ML), Internet of Things (IoT), big data, digital twins, blockchain, and CPS addressing safety, quality, traceability, and sustainability issues (Yadav et al. 2021a). The agri‐food supply chain deal faces sustainability challenges due to consumer demand for premium, safer food. Raising costs and regional economic effects continue. Life cycle assessment and reducing carbon footprint are crucial for sustainable SCM. Socioeconomic & environmental issues, such as household income, price fluctuations, health problems, food scarcity, and greenhouse gas emissions, impact the food industry. Collaborations between stakeholders and agricultural communities are essential (Chen et al. 2021; Abubakar et al. 2024). Climate change impacts crop productivity, yields, water resources, soil quality, and nutritional qualities. Inspections of food origin, quality, safety, nutritional qualities, and ethics have expanded as a result of free trade policies and the globalization of agri‐food markets (Tahir et al. 2024; Nsabiyeze et al. 2024). However, harsh policies, legislation, and trade agreements for safer agri‐foods have also led to issues such as traceability, banned chemicals, spoilage, and new insect vectors. The food supply chain management emphasizes food quality, but bioterrorism threats (intentional use of biological agents to cause harm, illness, or death in humans, animals, and plants), consumer confidence loss, increased pressure for regulations, and increased import from regions with less stringent food safety laws also pose serious concerns (Nurgaliyev et al. 2024; Abideen et al. 2021). IoT‐based provenance and tracking systems in agri‐supply chains are based on unified structures, causing issues like data integrity, manipulation, and single points of failure. Ensuring high confidence between cooperative parties is crucial for verified information. Although a third party is thought to be required for information security and quality, blockchain can act as a decentralized certificate authority, confirming transactions and providing tamper‐proof cryptographic information to any point in the chain upon request (Bhat et al. 2021). Early prototype models are being developed as food corporations work together to expedite the integration of distributed ledger technologies into supply networks for agri‐food. Blockchain‐powered models for supply chain product traceability are being implemented by researchers.

Nevertheless, early results are restricted to CPU load, latency, and network traffic (Dey et al. 2021). (Tian 2017) has extended its use to include HACCP‐based active food monitoring. However, there may be obstacles to the blockchain technology application in the supply chain and food processing industry. Making use of innovative technologies like blockchain and the Internet of Things, several research and development (R&D) communities are creating effective food chain traceability systems to enhance food quality and safety. These systems enable remote monitoring at each point of the supply chain (Surasak et al. 2019; Boye and Agha 2025). Blockchain technology has the ability to improve the effectiveness of data management and strengthen security protocols within supply chains that utilize the Internet of Things (IoT). By maintaining public keys and providing a graphical user interface (GUI) for user identity management, smart contracts exhibit increased resilience against malicious entities. Furthermore, blockchain can accelerate data management across various domains, including non‐repudiation, proof of ownership, information integrity, functional scope, access control, and data authentication. In response to diverse application needs, numerous smart contract platforms are currently under development (Gallersdörfer and Matthes 2021). All the entities involved in the supply chain can view, alter and confirm data due to the use of blockchains, which are transparent. This enhances the market share and increases the independence from the transportation brokers by enhancing the visibility and enabling the suppliers to market their capability and inventory. It gets rid of the middlemen and improves the coordination of business activities across enterprises by allowing the small suppliers and medium‐sized enterprises (SMEs) to transact with the retailers or the end customers (Khan et al. 2024; Wu 2018). 4.0 technologies can improve the intelligence, integration, data‐driven nature, agility, autonomy, and connectivity of the agri‐food supply chain (AFSC) system. Additionally, while there is ample documentation of 4.0 technology to enhance other fields, there are not many comparable applications in AFSC (Yadav, Singh, Raut, et al. 2022; Fatima et al. 2024). Therefore, research into the 4.0 technologies that are transforming AFSC is necessary. Technology enhances every AFSC step. Technology aids in precision farming, sustainable production methods, and assessing the caliber of raw material providers during manufacturing and processing stages. Furthermore, information from 4.0 technologies aids in multiple decision‐making processes at various AFSC phases (Yadav, Singh, Gunasekaran, et al. 2022). The distribution stage benefits from technology, real‐time monitoring, waste minimization, and creative logistical processes, such as sharing. Through the facilitation of information exchange and cooperation, 4.0 technologies assist AFSC in becoming more resilient & sustainable. 4.0 technology also facilitates supply chain coordination, which is necessary to maintain sustainability (Kayikci et al. 2022). Smart farming (SF) has emerged because of recent technological and agricultural method advancements, which have improved agricultural practice control as the overall framework is summarized in *Graphical Abstract*. Furthermore, as 4.0 technologies are closely tied to precision, smart farming, and 4.0 principles, these ideas might be applied to AFSC's transition to fulfill future food demands. In order to properly apply these technologies, it is necessary to comprehend their recent advancements (Núñez‐Merino et al. 2020). In addition, the researchers need to keep moving forward with their work in this field, record the difficulties the usage of these technologies presents and decide how to proceed with further study.

## 2 Methodology

### 2.1 Research Design

This review adopts a systematic literature review methodology to analyze the impact of blockchain, IoT, and AI on digital transformation in food supply chains. Peer‐reviewed articles (2017–2026) were sourced from Google Scholar, Scopus, Web of Science, industry reports (e.g., IBM Food Trust and Walmart case studies), and IEEE Xplore using keywords like “smart agriculture,” “blockchain food traceability,” and “AI‐driven logistics” as visualized in PRISMA flow diagram (Figure 1). Following the application of predefined search keywords related to supply chain and IoT, over 250 records were identified, of which more than 100 studies were selected after relevance‐based screening. Qualitative thematic analysis was applied to categorize findings into technological applications (e.g., blockchain for transparency, IoT for real‐time monitoring). Limitations include a bias toward recent studies due to rapid technological advancements as shown in the PRISMA Flow Diagram.

**FIGURE 1:**
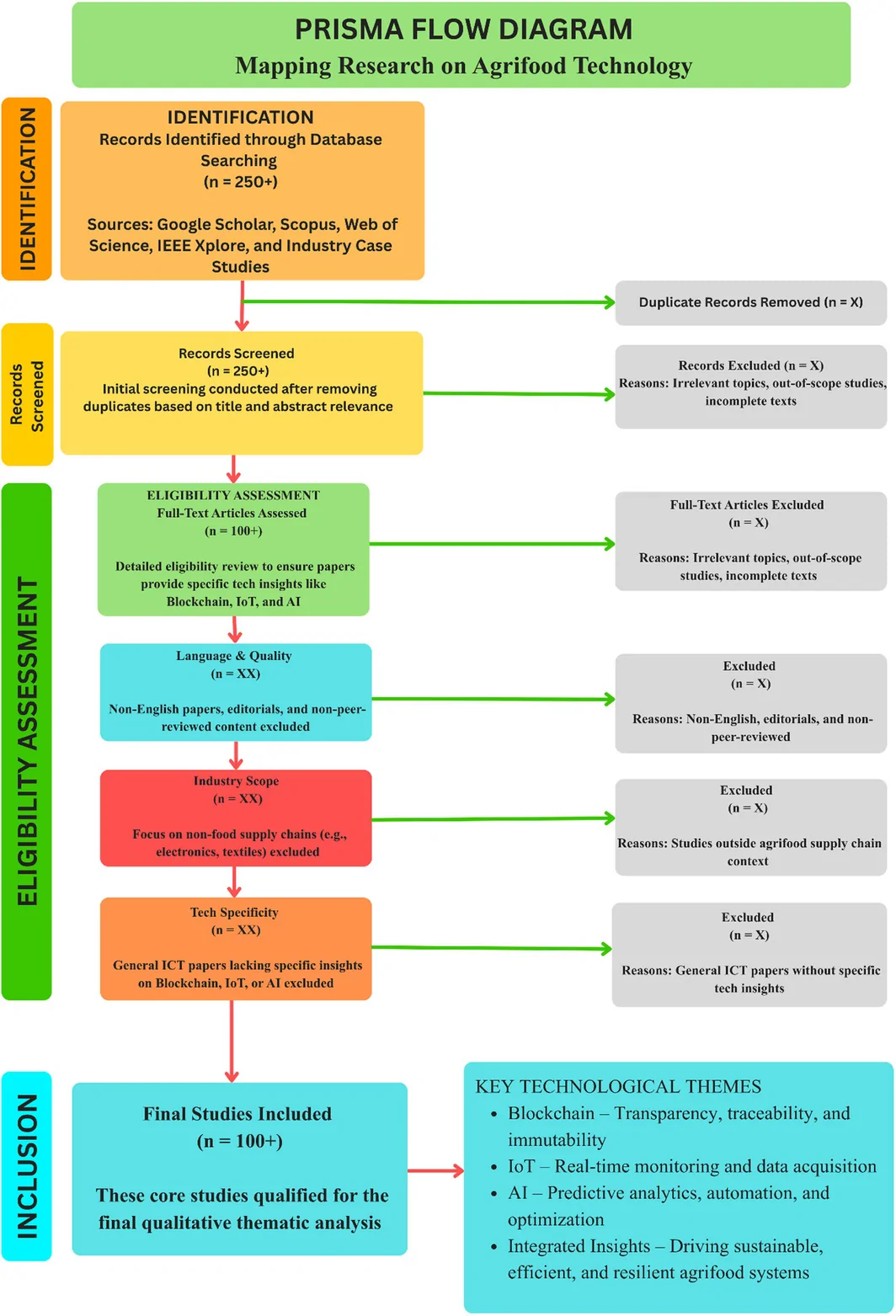
PRISMA flow diagram of mapping research on agri‐food supply chain.

### 2.2 Search Strategy

**TABLE 1:** The keywords reflect the integration of advanced technologies and data systems within contemporary food supply chain practices.

Technology	Keywords
Blockchain	Blockchain food traceability, smart contracts AFSC, decentralized SCM
IoT	IoT cold‐chain monitoring, RFID food logistics, real‐time sensors agriculture
AI	AI predictive analytics SCM, machine learning food waste, robotics harvesting

**TABLE 2:** Inclusion and exclusion criteria.

Criteria	Inclusion	Exclusion
Publication type	Peer‐reviewed article, case studies and meta‐analysis	Editorials, non‐English papers
Relevance	Focus on AFSC digitalization (farm‐to‐fork)	Non‐food SCM (e.g., electronics, textiles)
Technology	Studies on Blockchain, IoT and AI Applications	General ICT without tech specific insights

### 2.3 Analytical Framework

**TABLE 3:** Categorization of thematic analysis.

Theme	Sub‐theme	References/citations
Blockchain	Traceability, smart contract, security	(Yiannas 2018; Kamble et al. 2020)
IoT	Real‐time monitoring, automation, waste reduction	(Astill et al. 2019; Jagtap et al. 2021)
AI	Predictive analysis, robotics, NLP	(Davenport 2018; Rejeb et al. 2021)

### 2.4 Summary

This review article synthesizes findings from a wide array of scholarly papers, research studies, and industry reports addressing recent technological advancements in the food supply chain, with a focus on the integration of blockchain, the Internet of Things (IoT), and artificial intelligence (AI) as shown in Table 1. These emerging technologies have garnered significant attention for their potential to enhance transparency, traceability, operational efficiency, and food safety across all stages of the supply chain.

A critical assessment of prior research reveals substantial contributions by earlier scholars who developed conceptual frameworks and prototypes demonstrating the utility of blockchain in establishing secure and immutable transaction records that are shown in Table 4. IoT applications have been widely employed to enable real‐time environmental monitoring during production, storage, and transport, ensuring compliance with safety standards. AI techniques have facilitated improvements in areas such as predictive analytics, demand forecasting, and automated quality assurance.

**TABLE 4:** Technological footprints of blockchain, internet of things (IoT) and artificial intelligence (AI) in transforming the agriculture food supply chain.

Study	Technology	Key contribution	AFSC stage	Impacts
Yiannas (2018)	Blockchain	Walmart‐IBM mango traceability (2.2 s vs. 7 days)	Retail	Reduced recall cost by 30%
Astill et al. (2019)	IoT	RFID sensors for real‐time cold chain monitoring	Logistics	Cut food spoilage by 22%
Davenport (2018)	AI	AI‐driven demand forecasting for perishable goods	Production planning	Improved accuracy by 40%
Kamble et al. (2020)	Blockchain	Smart contracts for automated farmers payments	Procurement	Reduced payments delay by 90%
Jagtap et al. (2021)	IoT	Autonomous drones for warehouse inventory management	Storage	Increased efficiency by 35%
Rejeb et al. (2021)	AI	ML algorithms for dynamic pricing of surplus food	Distribution	Reduced waste by 18%
Galvez et al. (2018)	Blockchain	Tamper‐proof records for organic certifications	Farming	Increased consumer trust by 50%
Thirupathi et al. (2024)	IoT	Soil moisture sensors for precision irrigation	Production	Water savings of 25%
Raikwar et al. (2019)	Blockchain	Decentralized ledger for halal food traceability	Export/import	Compliance with EU/MENA Regulations
Malik et al. (2022)	AI	NLP for analyzing customer reviews on food quality	Retail	Improved recall response time
Torky and Hassanein (2020)	Blockchain	IoT‐Blockchain integration for seafood provenance	Logistics	Eliminated counterfeit products
Shahbaz et al. (2022)	IoT	Satellite imaging for crop health monitoring	Farming	Yield increased by 15%
Duan et al. (2024)	AI	Robotics for automated food picking	Harvesting	Labor costs reduced by 60%
Behnke and Janssen (2020)	Blockchain	QR codes for consumers facing product histories	Retail	Boosted sales by 20%
Surasak et al. (2019)	IoT	GPS enabled tracking for livestock movement	Farming	Diseases outbreak prevention

The advanced food supply chain, as explored in the reviewed literature, encompasses interconnected stages from primary production to final distribution as discussed in Table 2. At each stage, these technologies have played a pivotal role in minimizing risks, optimizing resources, and enhancing data‐driven decision‐making.

The collective impact of these technological interventions indicates a paradigm shift toward smarter, more resilient, and consumer‐centric food supply systems. This review highlights the growing importance of digital innovation in addressing challenges such as food fraud, sustainability, and global food security.

## 3 Overview of the Digital Transformation

Implementing digital technologies in the food supply chain necessitates a great deal of work from all stakeholders to get over potential roadblocks, which might include anything from technical problems (such as connection or storage requirements) to privacy and sensitive data protection (Annosi et al. 2021). The complex network that connects farmers, wholesalers, retailers, and consumers is undergoing significant disruption due to the application of digital technology. Transparency is being promoted, industrial sustainability is being increased, and long‐standing inefficiencies are being resolved.

### 3.1 Enhancing Transparency and Traceability

One of the biggest effects of the digital transformation in the food supply chain is the application of blockchain technology. From farm to fork, a product's complete route may be tracked, verified, and documented by all parties. This openness boosts consumer confidence by ensuring food safety, combating fraud, and encouraging ethical sourcing practices (Behnke and Janssen 2020). For example, consumers can access a product's origin, manufacturing processes, and quality certificates by scanning a QR code on it. Transparency in supply chains (SC) is seen favorably by society at large as well as by the SCM community. As a result, it is seen as essential to the pursuit of more liberating social and environmental policies and initiatives (Gold and Heikkurinen 2018). In other instances, partners do not necessarily view transparency as a good strategy. Misuse of trust or purposeful concealment of procedures and hazards may be the source of transparency's negative aspects.

### 3.2 Data Analytics to Optimize Operations

Digital transformation is also significantly influenced by advanced data analytics. Businesses can forecast demand, control inventory, and cut down on food waste by evaluating an enormous volume of data. Artificial intelligence (AI)‐powered predictive analytics aids in supply chain logistics, harvest schedule optimization, and weather pattern forecasting (Malik et al. 2022). In addition to cutting expenses, these insights guarantee that consumers will always have access to fresh products. Numerous publications discussed how to optimize decision support systems, costs, environmental effects, and routing issues using integrated modeling (Dutta and Shrivastava 2020).

### 3.3 IoT‐Powered Logistics Streamlining

The food supply chain logistics have been completely transformed by the Internet of Things (IoT). Swarm robots that move around items on rails can also be employed in this kind of environment. Additionally, application of robots includes product distribution, packaging, sorting, and transportation. To ensure the safety of their working environment, which may also require human labor, these mobile robots are outfitted with proximity sensors and warning systems. In production facility warehouses, drones can also be used as inventory control and monitoring (Dekhne et al. 2019). During transit, real‐time monitoring of storage parameters like temperature & humidity is made possible by smart sensors and networked equipment. This prevents spoiling and upholds quality standards by guaranteeing that perishable commodities stay fresh. Furthermore, real‐time cargo location updates are provided via GPS‐enabled tracking devices, enabling quicker and more effective delivery, as mentioned in Figure 4. Food goods and other resources can be tracked, traced, and monitored using some of the IoT applications that have been successfully deployed. This has enhanced efficiency and traceability (Jagtap et al. 2021). Automation primarily increases productivity by decreasing the time required to do jobs, which increases output and profit. Automation promotes accuracy, which in turn reduces errors, production requirements, waste, and boosts profit. A system's competitiveness in the market increases with its productivity (Jagtap et al. 2020).

#### 3.3.1 Enabling Farmers Through Smart Farming

Current developments in the Internet of Things facilitate the gathering of data on variables such as soil fertility, temperature, and weather. Crop web‐based surveys evaluate crop temperature, water quality, soil moisture content, and moisture status. Farmers may learn where, how, and how long to stay with the help of IoT. To keep an eye on home circumstances, remote sensor properties are utilized. IoT advancement might save expenses and promote typical expansion productivity (Thirupathi et al. 2024). Farmers are also becoming more productive and sustainable. With the help of drones, satellite imaging and Internet of Things devices. Precision agriculture allows farmers to better monitor the condition of their land, use water, and apply fertilizer. In addition to increasing crop yields, these technologies reduce resource waste, which helps to conserve the environment (Shahbaz et al. 2022; Ishfaq and Khalid 2025).

## 4 Current Challenges

Despite the revolutionary nature of digital technology adoption in the food supply chain, some challenges need to be properly addressed. One of the main challenges is that small‐scale producers and stakeholders may find it costly to adopt cutting‐edge technologies like blockchain, IoT, and AI (Galvez et al. 2018). Additionally, technological problems are often the consequence of the combination of numerous systems across multiple supply chain participants, making it more difficult to link and share data in a smooth way (Duan et al. 2024). Due to the increased susceptibility of the supply chain to cyber threats and violations, data security and privacy are also major issues. Subsequently, the utilization of digital platforms is increasing. Furthermore, if workers lack digital literacy, it could be more complicated for them to use these technologies effectively, which calls for large‐scale training programs. Lastly, firms must cultivate a culture of innovation and cooperation because skepticism about new approaches and resistance to change can hinder adoption rates. These problems need to be corrected if the food supply chain is to completely benefit from digital transformation.

## 5 Blockchain

Worldwide survival relies on agriculture, and expansion relies on strengthening food management, productivity, quality & marketing frameworks (Xu et al. 2020). Traceability & food safety are necessary components of this procedure(Duan et al. 2020). According to Raikwar et al. (2019) common definition of blockchain is; “Blockchain is a distributed ledger that keeps an ever‐growing list of data entries that every node involved checks.” Blockchain technology is a decentralized, unchangeable record of transactions that keeps users' identities and transactions safe. It is based on a cryptographic hash (Wamba and Queiroz 2020). It is very useful in the logistics of a supply chain, where tracking product make sure that perishable goods are always available and helps keep things from getting delayed. Blockchain technology can also be used for smart contracts to settle transactions and crop insurance schemes that don't need third parties (Torky and Hassanein 2020). Problems with agriculture and food supply chains, like inefficiency, low visibility, and absence of traceability, can affect food safety, reducing waste, compliance & operational costs (Kamble et al. 2020). Customer satisfaction and unexpected spikes in product demand are strongly reliant on supply chain management. Advances in centralized systems are crucial since traditional systems lack security threats, traceability, and transparency as discussed in Table 3 (Demestichas et al. 2020).

### 5.1 Core Features and Functional Mechanisms of Blockchain Technology in Supply Chains

By working as a public ledger over dispersed networks, blockchain technology addresses problems with information maintenance such as validity and verification. It increases product quality, lowers transaction costs, keeps a tamper‐proof record and offers security. Cryptographic techniques enhance consumer trust, which increases demand for products (Scuderi et al. 2019). Peer‐to‐peer networks help in verification, while blockchain‐related encryption techniques ensure that users and new blocks are legitimate (Akram et al. 2020) as shown in Figure 2. Because it is decentralized and empowers communication between entities without the need for a negotiator, it promotes trust and transparency in collaborative settings (Khan et al. 2020). All parties can reach an agreement thanks to the distributed ledger's decentralized structure, which guarantees traceability and immutability. In contrast to conventional databases, it cannot be changed or removed (Basnayake and Rajapakse 2019). By defining the logic of a contract between parties and imposing requirements on them, smart contracts can be put into place on the ledger. When utilized befittingly, the key components of blockchain technology—decentralization, immutability, security, and smart contracts—can provide significant advantages.

**FIGURE 2:**
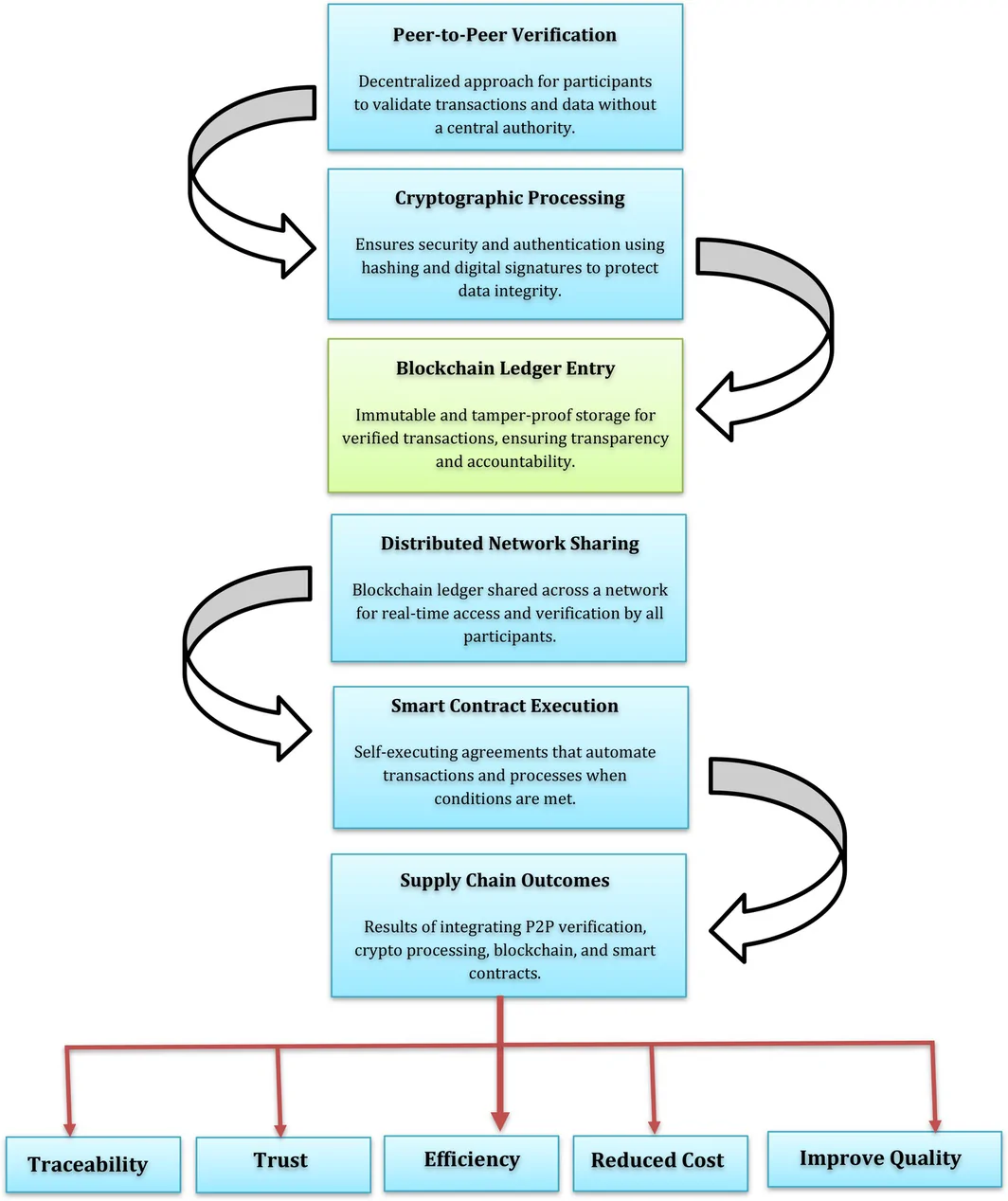
Peer‐to‐peer verification in supply chain management.

### 5.2 Decentralization

By promoting direct user‐to‐user transactions, decentralization facilitates information inequality & does away with the need for central authorities to ratify traditional transactions. Within a network, it guarantees that each authorized user has balanced power (Queiroz et al. 2020). Users support one another by keeping copies of documents, verifying transactions, and sharing the ability to view history at any time (Kamble et al. 2020). At all stages of the food supply chain, from suppliers of raw materials to consumers, product details can be documented as describe in Figure 2. Records are stored in copies whenever needed by a variety of stakeholders that are accessible (Hastig and Sodhi 2020).

### 5.3 Security

The Proof of Work (PoW) consensus method in blockchain technology protects data by requiring user approval for transactions. Hacking threats are reduced and supply chain failures are avoided by decentralization. Hacking occurs when the majority of users take control, which makes it harder to breach complicated networks (Reyna et al. 2018). Blockchain technology removes the risk of data theft & hacking in the food supply chain by protecting records and data.

### 5.4 Immutability

Blockchain enables validity and novelty by enabling authorized people to update and verify data. It is useful in food recalls and investigations because of its hard‐and‐fast property, which reduces the need for human intervention (Kamble et al. 2020). It ensures businesses can spot ingredients and track supplier chains, supporting fair trade, halal, and organic products. Immutability, however, does not ensure the veracity of raw data as shown in Figure 2 (Pournader et al. 2020). Blockchain encourages stakeholders to supply high‐quality information as the volume of transactions rises.

### 5.5 Smart Contract

The smart contract feature of blockchain technology can expedite transactions, increase confidence, and cut down on paperwork (Reyna et al. 2018). Unlike conventional supply chains, it can reduce processing time and personnel costs. Because smart contracts are based on agreements from all partners, lone users cannot make changes (Zhao et al. 2019; Qamar et al. 2025). By doing this, transaction risks are removed, supply chain transparency is raised, and efficiency is increased, clearly described in Figure 2. Maximum digitization, processing time savings, and cost reduction are all possible with blockchain, which will eventually give benefits to all parties that are involved (Kshetri 2018).

Blockchain technology offers a multitude of benefits, including enhanced security, transparency, traceability, food recall, enhanced trust, and ultimately enabling new business models and opportunities for innovation and growth.

### 5.6 Food Traceability

To handle their intricate supply chains, food industries employ a variety of computer and paper traceability solutions (Pearson et al. 2019). Private databases might impede stakeholder collaboration, and manual solutions can be laborious and prone to mistakes (Anwar et al. 2024; Hua et al. 2018). Complex supply chains can cost up to $93 billion, raise recall risks, and necessitate stakeholder participation. During food recalls, blockchain technology may increase confidence and traceability efficiency (Yiannas 2018).

It has been demonstrated that blockchain technology enhances food authenticity and traceability, lowering fraud and boosting confidence. Research by (Caro et al. 2018) and (Galvez et al. 2018) has shown that it can offer audit ability and transparency. In 2016, Walmart and IBM carried out a pilot project to employ blockchain technology for mango traceability, reducing the trace time from 7 days to 2.2 s (Yiannas 2018) as shown in Table 4. Blockchain also enhanced traceability in the Australian grain industry, according to Agri‐Digital and CBH Group.

### 5.7 Food Transparency

Big food brands often release partial information to benefit themselves, causing customers to lack knowledge about products and suppliers. Insufficient information can impact food security (Reyna et al. 2018) and customer trust, especially for halal food (Tieman and Darun 2017). Governments' power can be used for bribery and cover‐ups, as seen in the Sanlu milk scandal. Centralized supply chains struggle to achieve credibility and trust due to low transparency and visibility (Caro et al. 2018).

Information asymmetry is addressed and supply chain transparency is increased via blockchain's decentralization feature, which permits authorized users to conduct transactions and view history without the interference of a central authority (Queiroz et al. 2020). Its immutability characteristic guarantees that data is unchangeable and cannot be changed without informing every user. This makes it possible to quickly retrieve and update food product certifications, verifications, and product histories. Documents and records that are digitalized save time and reduce the possibility of errors and data tampering (Yiannas 2018). According to a 2016 study by Walmart and Tsinghua University, blockchain can effectively increase the validity and trust of information.

### 5.8 Food Recall

By enhancing productivity and focusing on food recalls, blockchain technology can build up the food supply chain. A clear understanding of the product flow and quick action are made possible by real‐time product information upgrades. Walmart can enhance shelf life, expedite inspection procedures, and track newly imported goods (Yiannas 2018). Narrowing recall ranges, identifying and isolating products from suppliers, and predicting increasing consumer demand are all possible with blockchain‐based traceability (Wang et al. 2019). Through recordkeeping in blockchain platform‐based water trade, blockchain technology can also result in sustainable water management (Zhao et al. 2019).

### 5.9 Challenges

Blockchain technology can modify the food supply chain, enhance productivity and reduce hazards. Before becoming extensively used, it must overcome some barriers, as it is still in its inception.

- Legislation and regulations need to be changed (Tse et al. 2017).
- The challenge of mandating that all parties embrace blockchain (Perboli et al. 2018).
- The possibility of raw data modification prior to uploading to the blockchain (Galvez et al. 2018).
- Scalability issues with technology (Pearson et al. 2019).
- Companies' depth of knowledge and comprehension is lacking (Kamilaris et al. 2019).

## 6 Internet of Things (IoT)

Combining items for data collection and transmission, the Internet of Things (IoT) is a smart, reliable, and quick information network. IoT includes wireless sensor networks (WSN), geographic information systems (GIS), global positioning systems (GPS), radio‐frequency identification (RFID), and more. With IoT sensors that collect data like voice, temperature, and humidity, IoT has the ability of automatic recording. IoT gives real‐time data collection for fresh food goods in supply chain management (SCM), which is used to identify the product quality in relation to the external environment (Astill et al. 2019), as shown in Table 4. IoT devices enhance the efficiency of information monitoring & gathering while also helping to the removal of human error.

### 6.1 Integration of Blockchain and IoT in Smart Agri‐Food Supply Chain Management

Blockchain and IoT permit smooth transactions and block‐by‐block data transfer tracing. Supply Chain Management (SCM) is now one of the current advancements in blockchain technology (Zhao et al. 2019). It can be successful if supply chain management (SCM) provides reliable insight on products and materials from the point of production to product delivery. Production firms may now achieve this insight, thanks to IoT, by utilizing smart supply chain management (SCM), which is IoT‐enabled to maximize production and allow efficient transmission. The shipping service for customers is also being enhanced through smart SCM as shown in Figure 3 (Umamaheswari et al. 2019). Several IoT‐enabled sensors work in different phases of the supply chain in these systems to extend features like real‐time supply chain monitoring, which may be operating to keep an eye on the attributes of raw materials and components. These sensors detect and respond to changes in an environment in real time.

**FIGURE 3:**
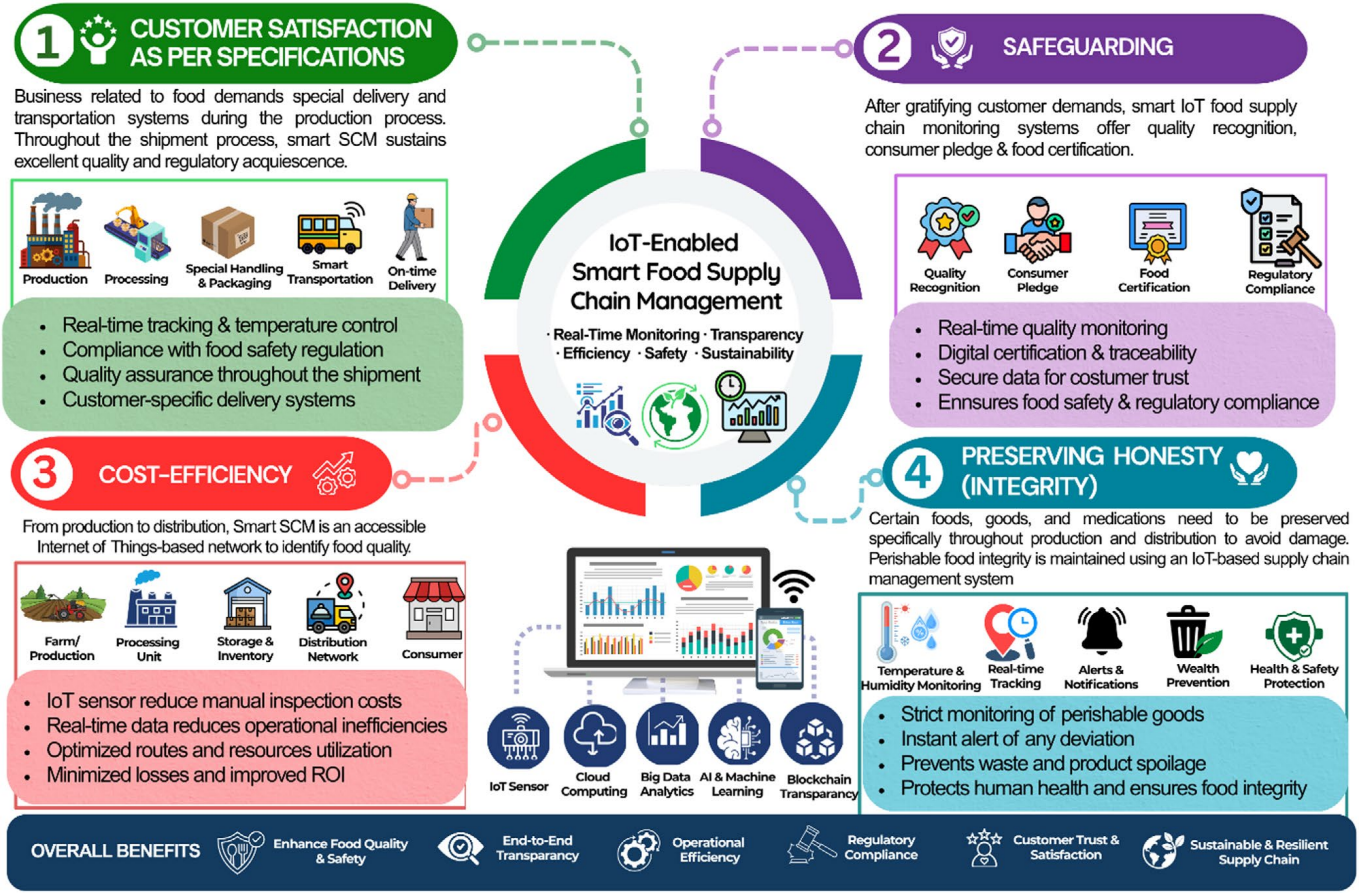
Identification of key challenges in agri‐food supply chains and how Internet of Things technologies are employed to mitigate them.

- Customer satisfaction as per specifications: Businesses related to food that demand special delivery and transportation systems during the production process. Throughout the shipment process, smart SCM sustains excellent quality and regulatory acquiescence as shown in Figure 3.
- Safeguarding: After gratifying customer demands, smart IoT food supply chain monitoring systems offer quality recognition, consumer pledge & food certification briefly describe in Figure 3.
- Cost‐efficacy: From production to distribution, Smart SCM is an accessible Internet of Things‐based network to identify food quality clearly shown in Figure 3.
- Preserving Honesty: Certain foods, goods, and medications need to be preserved specifically throughout production and distribution to avoid damage. Perishable food integrity is maintained using an IoT‐based supply chain management system. Smart supply chains use strict monitoring and alarms to prevent waste and protect human health as shown in Figure 3.

### 6.2 Integration of AI, IoT, and Blockchain in Smart Supply Chain Management: Opportunities and Challenges

Artificial intelligence (AI) technologies that address trust, traceability, security, and collaboration challenges in supply chain management (SCM) are linked to blockchain technology. Contemporary supply chains are made up of several components, such as automation, gadgets, transportation, and services. Traditional supply chains function independently at planning, purchasing, distribution, marketing, and manufacturing phases. Direct connections between these parts allow IoT devices to send data to a cloud for storage (Chang et al. 2022). Scalability, interoperability, auditing, security, efficiency, and quality are all benefits of integrating blockchain technology with IoT. A blockchain‐based, self‐organizing, Internet of Things‐based agricultural system was unveiled. The position and status of events can be tracked by cloud‐based technology, including Internet of Things devices, which allows for real‐time inventory management and planning forecasts as discussed in Table 3 (Thirupathi et al. 2024). But there are obstacles like data manipulation, security transaction techniques, and even IoT device hacking. Despite offering supply chain integrity and real‐time insight, blockchain technology has drawbacks like data manipulation and security concerns.

### 6.3 Food Composition, Quality and Safety Monitoring

Several awareness campaigns about healthy lifestyles and technical developments in food science have recently raised public knowledge of the quality and composition of food products (Vangala et al. 2020). In addition, businesses must clearly label food goods and maintain quality control due to new global food standards. Labeling standards play a vital role in determining whether scientific experiments are appropriate for a given problem. A growing number of consumers worldwide are demanding assurances that the ingredients and place of origin of their food match the information on the label (Yu et al. 2022). This explains why consumers, manufacturers, and government agencies have viewed food products' authenticity as important quality requirements. The quality, composition, and assessment of food products are therefore rapidly advancing thanks to various IoT sensors that used UV–visible–near infrared spectroscopy (Mendes and Duarte 2021), DNA‐PCR procedures (Dronina et al. 2021), stable isotope measurement (IRMS) (I. Fernando et al. 2024), mass spectrometry (Núñez‐Carmona et al. 2021), separation techniques (Mosallanezhad et al. 2023), and big data. As a result, distributed ledger technology, IoT, big data, micro‐fabrication, and the shrinking of spectroscopic instruments have all advanced. In general, the Internet of Things (IoT) based on UV–visible near‐infrared spectroscopy, when combined with blockchain and big data analysis, offers a robust and important technique for managing authenticity and variation in the smart agricultural field.

### 6.4 Food Safety Across Supply Chain

From farm to fork, food safety appeared as a worldwide concern for all parties involved. Fresh agricultural goods have been the source of numerous foodborne outbreaks in recent years. In order to safeguard the public health, a significant amount of agricultural produce is dumped (Kumarathunga 2020). Given that the world's food needs are predicted to increase by 50% between 2020 and 2050, current methods of preventing foodborne illnesses from endangering the public health may not be viable options in the future (Mirabelli and Solina 2020; Qureshi et al. 2024). Among the many advantages that Information and Communication Technology (ICT) can add to food safety in the prospectus and parallel monitoring of agri‐food products throughout storage and distribution (Torky and Hassanein 2020), are RFID tags, GPS, and QR codes that function as advanced labeling that strongly matches the cloud and enhanced traceability as shown in Figure 4. Blockchain technology and the internet of things have the ability to improve food inspection systems, and they function in tandem by using AI‐based big data analysis, such as providing a timely response to food safety emergencies (Singh and Singh 2020). It is anticipated that the internet of things will help implement a thorough approach to food management, where important catalysts such as economics, human behavior and environmental change may be combined to evoke food safety risks and blockchain will increase privacy and data security (Hu et al. 2021).

**FIGURE 4:**
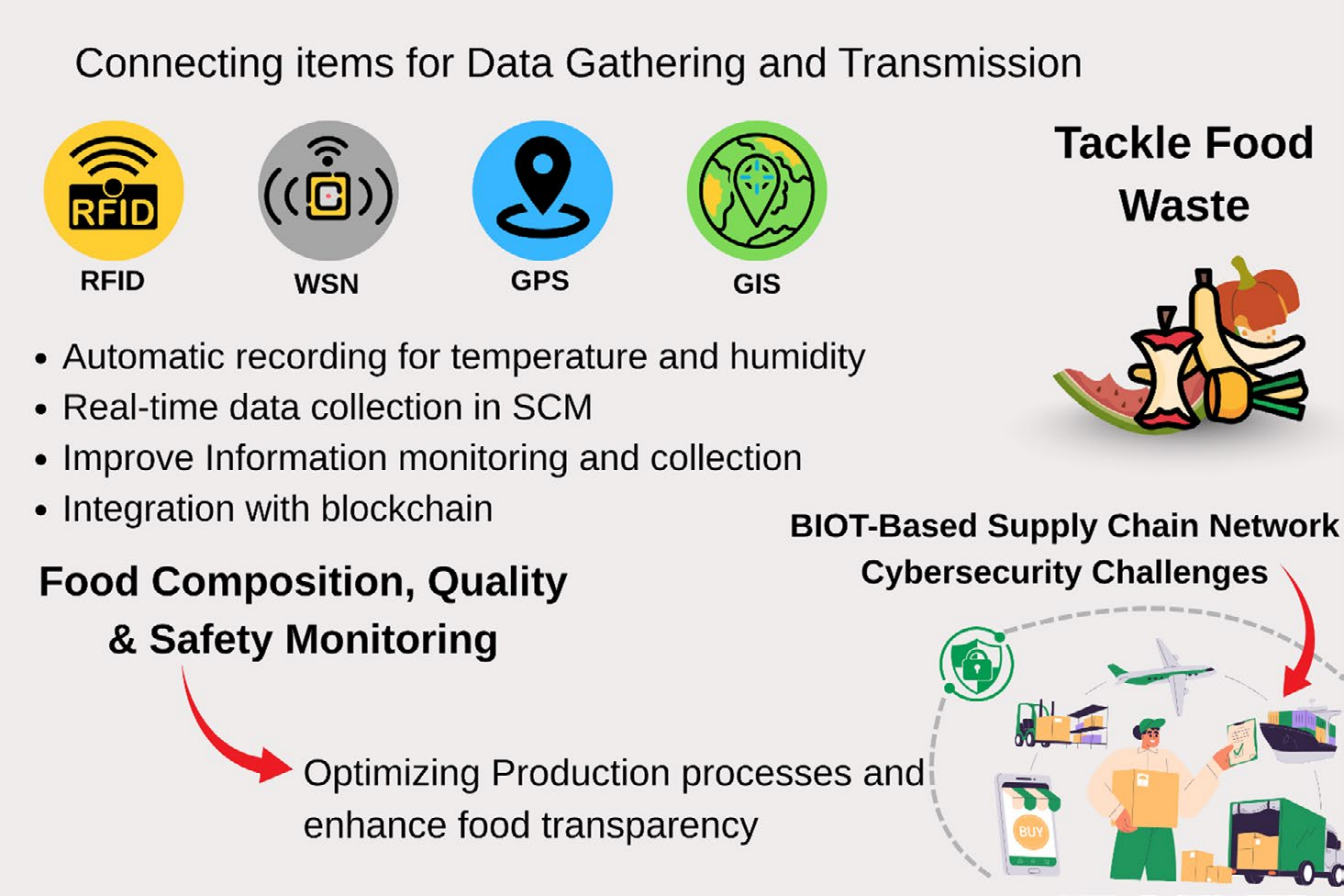
The adoption of IoT in agriculture offers benefits such as predictive analytics, reduced waste, and improved supply chain transparency.

#### 6.4.1 Tackle Food Waste

Food loss arises at different points in the food supply chain, including harvesting, post‐harvest management, storage, preparation, distribution, and consumption (Salihoglu et al. 2018). Food waste includes losses brought by illnesses and pests prior to harvest or extreme weather conditions. This leads to economic losses for farmers and shortages and increased costs for customers. Technology‐based solutions like blockchain, IoT, and big data are used to control food waste, increase shelf‐life, make dynamic price adjustments, and monitor food waste at restaurants and retail stores as described in Figure 4. Blockchain‐based supply chains provide transparency into food production, while IoT devices optimize production processes and enhance quality (Baralla et al. 2019).

### 6.5 BIoT‐Based Supply Chain Network Cyber Security Challenges

Multiple parties like businesses, governments, and nations are involved in supply chain networks, which make them complex. They face challenges such as trust establishment, cyber‐attacks, cargo theft, and counterfeiting. The complexity of supply chains increases vulnerability to security threats, such as fraudulent replacement and tampering (Dong et al. 2019). The future implications of IoT are evident in its applications in various fields, such as the agriculture industry. A multidisciplinary strategy that addresses both hardware and software is required due to the growing dangers of security assaults on smart farm infrastructure. There are three tiers of security issues in an Internet of Things network: the application layer, the network layer, and the perception layer (Hassija et al. 2020), as shown in Figure 3. At the application layer, security concerns include user privacy, safe computing, and encrypted data analysis. IoT devices can access the network using cellular networks with advanced security protocols. In the perception layer, threats include open insecure ports and vulnerabilities, weak login and password mechanisms, and inadequate network and device encryption methods (Carbone et al. 2018).

## 7 Artificial Intelligence (AI)

A subset of machine learning known as artificial intelligence involves computers simulating human cognitive processes (Chen et al. 2020) as shown in Figure 5. The AAAI (Association for the Advancement of Artificial Intelligence) defines AI as “promoting scientific knowledge of the mechanisms underlying intelligent behavior and thought & their embodiment in machines.” “AI, a field of computer science created to produce this intelligence, is defined as the intelligence of computers and software,” according to a popular textbook by Russell and Norvig (Soleimani 2018). The goal of artificial intelligence is to comprehend intelligent beings. Artificial intelligence is the capacity of robots to make judgments based on a series of actions and learn from experience (AI) (Duan et al. 2019).

**FIGURE 5:**
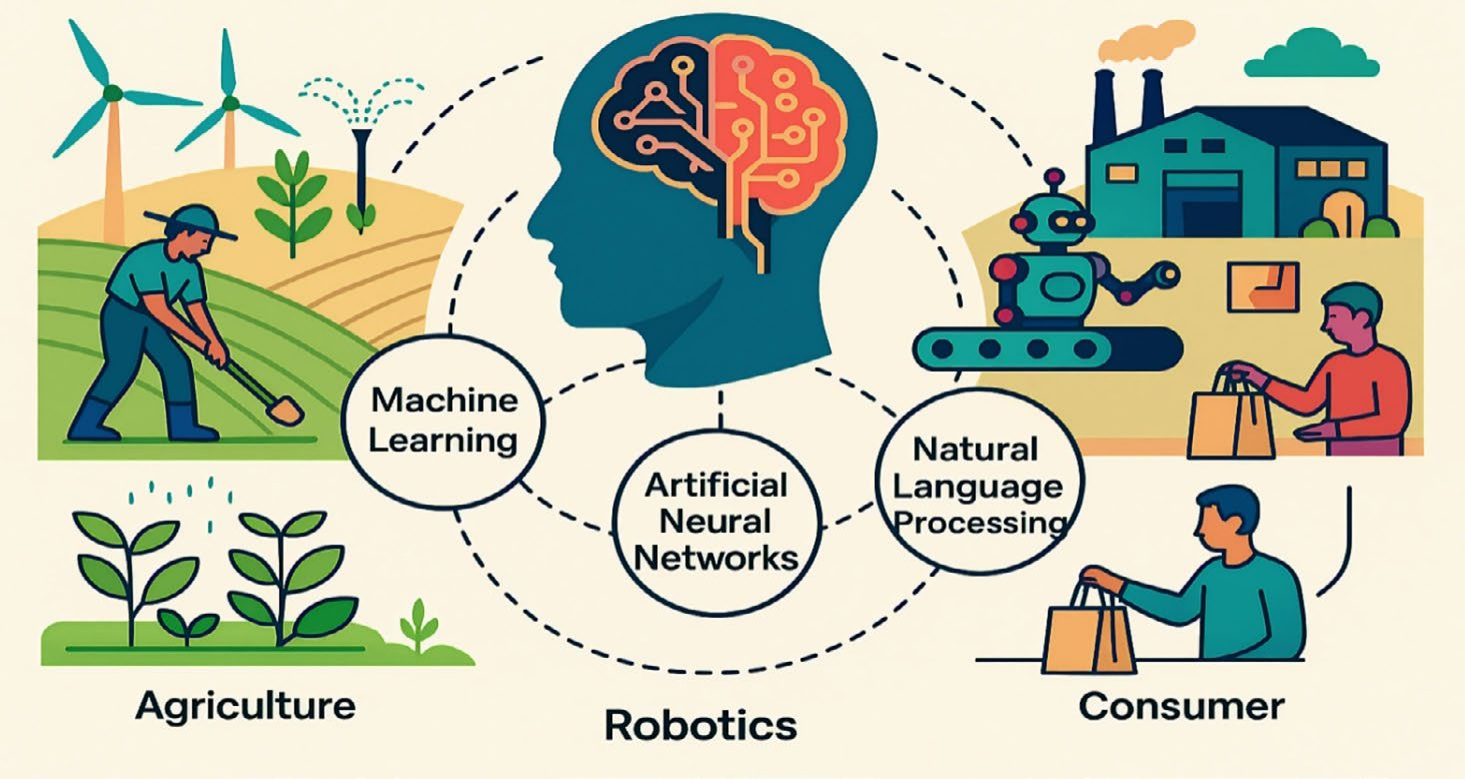
AI integration of artificial intelligence (AI) technologies within agriculture and food supply chain to enhance efficiency, traceability and data‐driven decision‐making.

### 7.1 Artificial Intelligence in Supply Chain Management: Concepts, Technologies, and Applications

Artificial intelligence (AI) is a fast‐emerging field in computer science due to recent developments in deep neural networks, convolutional neural networks, mathematical optimization techniques used in operations research, constraint programming, and various numerical methods (Jan et al. 2023). These advancements have made it possible for computers to carry out tasks that were previously only possible for humans. Recently, a variety of industries have become more interested in the possible uses of artificial intelligence (AI) technology (Dubey et al. 2020). These days, AI affects supply chain management (SCM) holistically as mentioned in Figure 6. For technological reasons, SCM is the starting point for the development of machine learning and most intelligent technologies. AI adoption in supply chain management (SCM) is being done for business purposes since it can improve consumer goods and services, boost supply chain visibility and transparency, and boost customer satisfaction. Businesses that have attempted to employ AI in supply chain management include Amazon, Walmart, Philips, eBay, and others (Dwivedi et al. 2021). According to Davenport (2018), artificial intelligence (AI) technically necessitates enormous volumes of data, extensive data processing, and (mostly) state‐of‐the‐art statistical techniques. Natural language processing (NLP), machine learning (ML), cyber‐physical systems (CPS), expert systems, visual processing, audio processing, neural networks, robotics, and other techniques are used in the numerous subfields that make up artificial intelligence (AI) (Duan et al. 2019).

**FIGURE 6:**
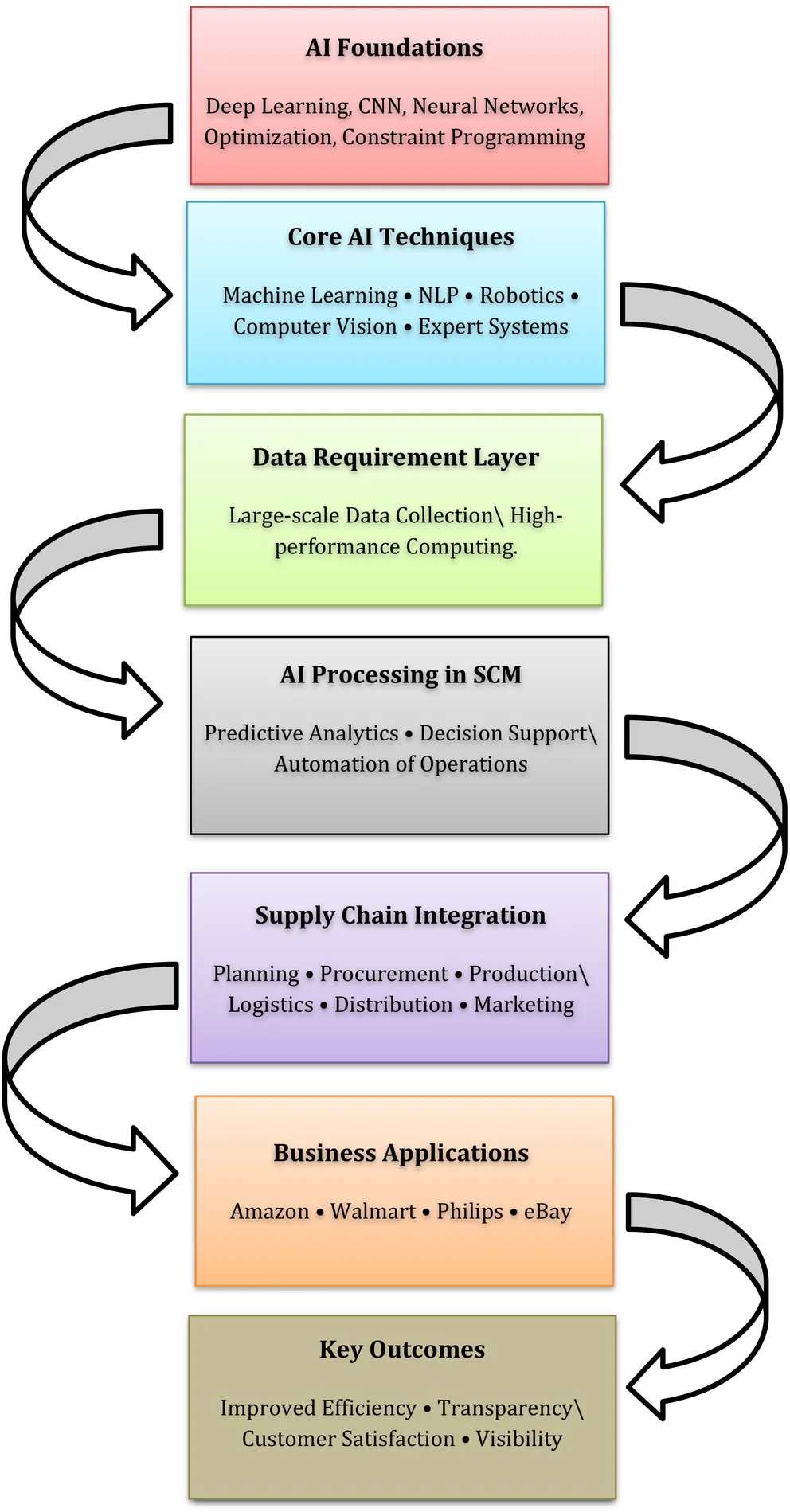
AI integration in supply chain management.

The following list of the most popular AI techniques has been chosen and is presented:

### 7.2 Machine Learning (ML)

One of the most important AI technologies is machine learning (ML), which allows machines to process unstructured information in addition to data (Rejeb et al. 2021). In addition to learning from data, machine learning (ML)‐based systems can recognize patterns in vast amounts of samples, make judgments based on structured feedback, and then carry out tasks independently. Eventually, with little assistance from humans, these systems can continue to enhance their functionality and problem‐solving abilities (Brynjolfsson and Mcafee 2017). Astronomy, agriculture, and medicine have all seen tremendous advancements because of machine learning, which has made it possible to analyze vast amounts of data and find intricate patterns (Ezugwu et al. 2023) as shown in Figure 5.

All “demanding” to “subsequential” operations, including agricultural cultivation, crop production, processing, testing, packing, storage, and marketing, are covered by the Agri‐Food Supply Chain (AFSC). Strategic, tactical, and operational decision‐making procedures as well as managerial activities are necessary for AFSC operations to be effective (Saetta and Caldarelli 2020).

Compared to other supply chains, AFSC has more complicated features due to its seasonal, varied, and perishable character as well as the rising consumer consciousness regarding the quality and safety of products. Four activities are included in AFSC: food processing at processors (producers), distribution at distributors, production planning and control at suppliers, and consumption at retailers/customers (Sheriff and Aravindhar 2024).

### 7.3 Artificial Neural Networks (ANN)

Another essential AI method that draws inspiration from the way the human brain functions is neural networks (Katal and Singh 2022). A model was served as a biological nervous system. In order to differentiate features, identify patterns, group things, analyze ambiguous or abstract information, and learn from past instances and experience, it makes use of an interconnected network of computer memory (Sharma et al. 2020). A sophisticated sort of machine learning algorithm is called an ANN. It deals with big data sets and calls for specialized computer architectures and higher processing capacity (Brynjolfsson and Mcafee 2017). Medical image processing, machine translation, and autonomous driving are just a few of the domains where deep neural networks, also referred to as deep learning, have shown remarkable performance.

From modest garden settings to big commercial farms and regional monitoring systems, artificial neural networks (ANNs) in agriculture are incredibly scalable. Depending on the data and processing capacity available, they support a variety of activities, from localized disease detection to large‐scale yield predictions. They are useful in a variety of agricultural applications due to their error tolerance, data‐driven learning, robust prediction performance, and capacity to capture intricate correlations (Soleimani et al. 2026; Shahbaz and Ali 2025).

The foundation of AI techniques is computer‐aided systems that can be trained with past data. Neural networks and expert systems are the most common AI applications in SCM issues.

The ability of ANNs to do away with the necessity for formulas in decision‐making processes is a significant advantage (Soori et al. 2024). ANNs can more effectively handle the uncertainty and complexity of decision‐making situations thanks to this capability. Although ANNs are employed in many other fields due to their capacity to simulate human associative memory, their usage in SCM problems was restricted to a small number of problem types. An ANN is a potent modeling tool that aids in our comprehension of intricate input/output trade‐offs (Yin et al. 2025).

For applications like disease diagnosis, yield prediction, and robotic navigation, deep learning (DL), which is based on ANNs, efficiently pulls characteristics from massive agricultural datasets. By learning directly from raw data, Convolutional Neural Networks (CNNs), Region‐based Convolutional Neural Networks (R‐CNNs), and Recurrent Neural Networks (RNNs) increase accuracy and decrease the need for manual feature engineering (Wang 2024).

Two important DL models utilized in agriculture are CNNs and RNNs. Applications like crop‐weed separation, fruit maturity evaluation, and plant part identification are made possible by CNNs' superior performance in spatial tasks like classification, object recognition, and image segmentation. However, they are sensitive to visual modifications and have no memory for temporal data. In order to properly simulate sequential data for activities like plant development and monitoring, RNNs work in tandem with CNNs by storing previous knowledge (Ghalehkhondabi 2026). When combined, these models substantially improve AI‐driven vision systems, drastically enhancing agricultural decision‐making, efficiency, and precision.

### 7.4 Natural Language Processing (NLP)

One of the most well‐known branches of artificial intelligence is natural language processing (NLP), which studies the interaction between computers and human language (Maulud et al. 2021). NLP is the process of using past speech or textual patterns to extract meaning or information (such as entities, places, subjects, sentiment, etc.). This is accomplished either by statistically analyzing words or phrases (statistical natural language processing) or by using ontologies and semantic analysis (semantic natural language processing, or decomposition and links between words and phrases) (Davenport 2018).

By using natural language processing (NLP) in topic modeling, businesses can examine consumer input and identify recurring themes that might point to areas in their supply chain that require improvement. In this situation, the Latent Dirichlet Allocation (LDA) technique is frequently employed. Improved operational efficiency, lower costs, fewer risks, and higher profitability can result from effective supply chain management that is bolstered by insights from topic modeling (Aslam and Calghan 2023). Companies can gain more detailed insights than more generic analytics by comprehending specific consumer discourse about things like supplier connections, product quality, and delivery timetables. Furthermore, monitoring patterns in topic prevalence aids in spotting new concerns, including an increase in conversations regarding product damage, which may indicate handling or packing difficulties that require attention for long‐term expansion and client satisfaction (Ai and Aslam 2023).

By pinpointing areas for improvement, businesses can achieve significant advantages in supply chain management. This knowledge makes it possible to prioritize important issues and allocate resources effectively, resulting in focused improvements that tackle supply chain problems and promote more productivity and better results. Additionally, automatic classification of textual input into predetermined categories is made possible by text classification, a crucial research task incorporating machine learning algorithms. This approach facilitates effective data organizing and retrieval for applications like sentiment analysis and spam screening (Ferrari et al. 2017). Using techniques like Naive Bayes, Support Vector Machines, and Neural Networks, labels are assigned based on content throughout the text classification process. Improved coordination and cooperation among stakeholders are key components of effective supply chain management, which leads to increased customer satisfaction, cost savings, communication, and transparency (Holloway and Holloway 2024).

Businesses have found that Supply Chain Management (SCM) can be transformed by incorporating Natural Language Processing (NLP) techniques, particularly sentiment analysis. This method provides insights into customer happiness and identifies opportunities for development by using machine learning algorithms to gather and analyze subjective and emotional data from customer comments (Houda 2024). As a result, businesses may improve customer experience and supply chain performance by making data‐driven, well‐informed decisions.

### 7.5 Speech Processing (SP)

SP is the process of converting voice into digital signals by means of digital signal processing techniques. The voice‐directed system is implemented using speech recognition and other data‐collecting technologies. This technology gives consumers instructions through audio prompts. Additionally, users can reply by verbally verifying to the system that tasks have been completed (Levy 2018). Analyzing speech data to extract distinctive emotional features and classifying emotions using suitable models constitute the first stage in speech emotion recognition. The speech is digitally encoded, segmented, and divided into frames using header information since computers cannot process continuous speech signals (Li et al. 2022). These speech signals are difficult to identify and interpret; time‐domain and frequency analysis are frequently used, and a combination of model‐based and nonmodel‐based patterns is usually needed (Razavi et al. 2016).

Bell Labs' Audreyi system, which could recognize Arabic numerals, marked the beginning of the development of intelligent voice technology in 1952. The Hidden Markov Model (HMM) and Artificial Neural Networks (ANN), which improved voice recognition capabilities, were introduced in the 1980s. Intelligent voice systems have advanced to almost human‐like recognition with the incorporation of contemporary AI technology, moving from simple hearing to precise comprehension (Xu and Xia 2023). Virtual assistants like Siri and Xiao Ai are examples of applications in daily life that enhance human‐computer connection, particularly for the elderly and disabled. Intelligent voice technology is used in agricultural Q&A systems, livestock farming, and unmanned aerial vehicles (UAVs) to greatly increase production and solve manpower shortages. Researchers have created customized Q&A systems that leverage knowledge graphs to provide prompt answers on subjects like livestock illnesses and pest control (Yexin 2024). Sustainable pest management approaches are increasingly important for maintaining food safety and reducing reliance on conventional chemical pesticides. Essential oils have demonstrated acaricidal activity against *Dermanyssus gallinae*, suggesting their potential as alternative or complementary approaches for pest control in poultry production (Ratajac et al. 2024).

In 1952, the development of intelligent voice technology began with Bell Labs' Audreyi system, which could recognize Arabic numerals (Xu et al. 2025). The 1980s saw the introduction of Artificial Neural Networks (ANN) and the Hidden Markov Model (HMM), which enhanced voice recognition skills. With the use of modern AI technologies, intelligent voice systems have progressed from basic listening to accurate comprehension, reaching nearly human‐like recognition (Xinhe et al. 2025). Examples of everyday applications that improve human‐computer interaction include virtual assistants like Siri and Xiao Ai, especially for the elderly and disabled. Unmanned aerial vehicles (UAVs), animal farming, and agricultural Q&A systems all integrate intelligent voice technologies to significantly boost output and address labour shortages. In order to deliver quick responses on topics like pest control and cattle illnesses, researchers have developed specialized Q&A systems and used knowledge graphs (Lingfei et al. 2022).

### 7.6 Robotics

The diverse field of robotics includes computer science, mechanical engineering, electrical engineering, information engineering, and other fields (Ness et al. 2023). Space exploration and environmental catastrophe cleanup are two examples of applications for these robots in hazardous or inaccessible environments. AI gives robots the ability to make judgments instantly, adjust to changing conditions, and work effectively and safely with people (Soori et al. 2023), as shown in Figure 5.

The adoption of these technologies is still relatively new in comparison to other economic sectors; this is especially true when one considers the significant technological gaps that agribusinesses around the world face, the majority of which do not use precision agriculture that discuss in Table 7 because they lack access to opportunities or are ignorant of these tools (Khanal et al. 2018). On the other hand, it is feasible to determine that the great majority of agricultural items produced globally are produced using conventional methods, missing out on several technologies, including crop planning and irrigation. This reality creates a complicated picture for the application of AI in the industry, particularly when it is noted that agriculture is practiced unevenly and for subsistence in developing countries, making it challenging to use efficient algorithms to assess the state of the industry in those countries (Ramirez et al. 2024).

A major area of study is still achieving high performance in agricultural robots, which is made possible by improvements in identification and positioning hardware and software. Soil preparation, planting, weeding, harvesting, and yield assessment are some of the current uses (Bai et al. 2023). Agricultural robots can be categorized according to several factors, including development stage, application, sensor presence, and locomotion system. There is a noticeable trend toward 4WD robots that use RGB cameras to remove weeds. The exorbitant cost of technology, however, is a major obstacle that separates farmer desire from robot development (Pitla and Brumm 2020). Furthermore, further research is required to fully understand the safety, cultural, and socio‐political ramifications of robotic integration in agriculture. Numerous obstacles, both general and task‐specific, confront agricultural robots (Oliveira et al. 2021). Route design, safety concerns, especially human detection, and robot fleet administration are examples of general obstacles as shown in Figure 7. The needs of crop structure, the identification and categorization of crops or pests, and the accurate application of agricultural inputs are examples of activity‐specific problems (Sara et al. 2024). The hunt for the best solutions in vision systems, robotic actuation, navigation in semi‐structured environments, battery technology, and intelligence systems needed to manage the robotic platform and its tools is the root cause of many of these difficulties (McPhee et al. 2020).

**FIGURE 7:**
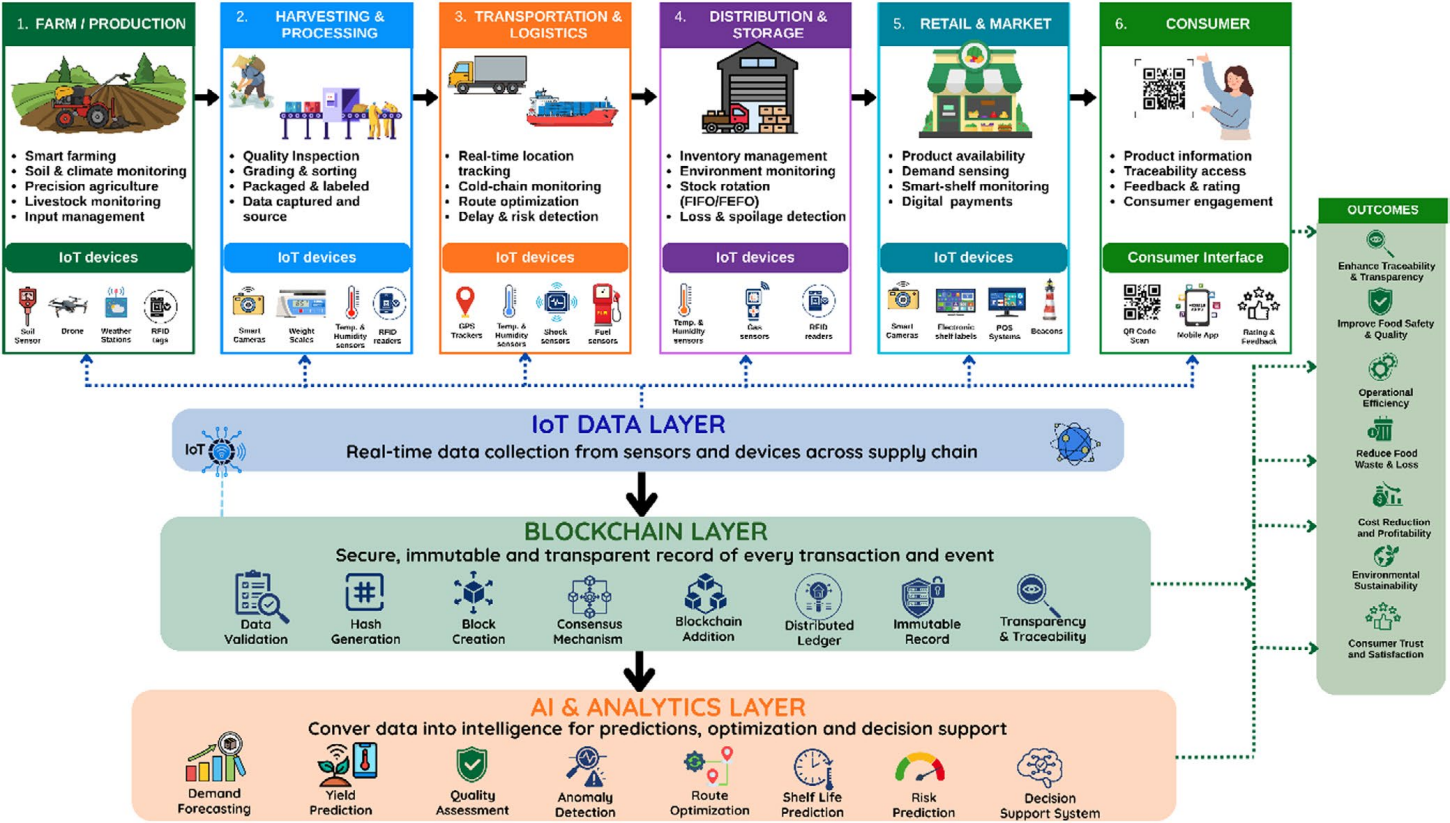
Integrated framework of blockchain, internet of things (IoT), and artificial intelligence (AI) for sustainable agri‐food supply chain management.

#### 7.6.1 Agricultural Robotics for Environmental Sustainability and Decarbonization

The environmental sustainability of using agricultural robots is another crucial factor. It should be emphasized that robotics will be crucial in decarbonizing agricultural production systems, as was mentioned in the Farm to Fork approach. Large‐scale agricultural robot use on farms can play a significant role in reducing compaction by lightweighting robotic machines (Chlingaryan et al. 2018); optimizing crop nitrogen use and lowering N_2_O emissions; reducing farm waste, including losses from pests and disease, by using artificial intelligence and machine learning (Liakos et al. 2018); and reducing carbon emissions by using electrified robotic vehicles (Rose et al. 2021). It becomes crucial to focus on the integration of technical systems with social and ecological ones during the design, manufacture, and use of robotic technology in order to accomplish these aims.

## 8 Integration of Blockchain, Internet of Thing (IoT) and Artificial Intelligence (AI) for Sustainable Agri‐Food Supply Chains

Table 5 analyses Blockchain, IoT, and AI in the context of agri‐food supply chains based on performance parameters including traceability, monitoring, decision assistance, security, cost, and scalability in order to summarize the examined literature. No single technology can solve every problem on its own, according to the report. Blockchain has excellent security and traceability, but it is expensive. IoT offers scalability and real‐time monitoring, yet it has cybersecurity flaws. AI provides excellent decision help, but it needs high‐quality data and processing power (Patel and Sureja 2025). In the end, combining these technologies produces a framework that improves agri‐food supply chains' sustainability, safety, efficiency, and transparency as discussed in Table 6.

**TABLE 5:** Comparative evaluation of blockchain, IoT, and AI technologies in agri‐food supply chains.

Criteria	Blockchain	IoT	AI
Traceability	Excellent	Moderate	Moderate
Real‐time monitoring	Low	Excellent	Good
Decision support	Low	Moderate	Excellent
Security	Excellent	Moderate	Moderate
Implementation cost	High	Moderate	High
Scalability	Moderate	High	Moderate

**TABLE 6:** Key research gaps and emerging challenges in digital transformation of agri‐food supply chains.

Research gap	Critical issue
Limited AI–Blockchain–IoT integration	Most studies investigate individual technologies rather than integrated architectures
Interoperability	Different platforms, sensors, databases, and blockchain networks often cannot communicate seamlessly
High implementation costs	Expensive infrastructure can restrict adoption by SMEs and smallholder farmers
Data governance and privacy	Sharing agricultural and commercial data creates ownership, privacy, and security concerns
Limited Explainable AI (XAI)	Complex AI models may provide accurate predictions but lack interpretability
Lack of standardization	Absence of common protocols limits large‐scale deployment
Insufficient real‐world validation	Many proposed systems remain at conceptual or laboratory/prototype stages
Limited developing‐country evidence	Rural connectivity, infrastructure, skills, and affordability require greater research attention
Limited sustainability assessment	Few studies comprehensively evaluate environmental, economic, and social impacts over the technology life cycle

## 9 Trends, Challenges and Future Agenda in Agri‐Food Supply Chain

A major worldwide concern that involves many parties and necessitates safe, high‐quality operations is the viability of international agricultural food supply networks (Niu et al. 2022). This chain's vulnerability has been brought to light by the COVID‐19 epidemic, necessitating digital transformation (Iqbal et al. 2025; Hobbs 2021). Big data and other digital technologies are necessary to enhance agricultural resilience, maximize resource utilization, and adapt to market fluctuations (Carter et al. 2021). The deployment of digital technology in these supply chains has drawn the attention of researchers due to these difficulties, which has encouraged scientific study in this area.

The following are the current areas of concentration while reviewing the literature to comprehend research advancements in the sustainability of agricultural food supply chains enabled by digital technologies:

1. Developing a smart agricultural food supply chain with big data: Cravero et al. (2022) offer information on many kinds of data utilized in agro‐food big data, as well as the primary issues and patterns associated with it. Big data technology is used by (Shen et al. 2022) to build very effective agro‐food recommendation algorithms. Donaghy et al. (2021) talk about how big data is likely to be used in agriculture and food supply chains for dynamic risk management.
2. In the agricultural food supply chain, blockchain‐enabled traceability: Bhat et al. (2021) suggest an agro‐SCM‐biot architecture. In their 2023 study, Srivastava et al. (2023) examined how blockchain technology might be used in food supply systems and agriculture. Mirabelli and Solina (2020) investigate how blockchain technology might be used to track food supply networks and agriculture.
3. Examine research on how digital technology might strengthen agricultural food supply systems. Yadav, Singh, Raut, et al. (2022) carry out a thorough analysis of how industry 4.0 technologies are being used in food and agriculture supply chains. A review of research on the advantages, difficulties, and potential developments of artificial intelligence in agriculture and food supply networks was carried out by (de Oliveira and Silva 2023). A survey of the literature on the use of digital twins in the food supply chain is presented by (Henrichs et al. 2021). Furthermore, other researchers have demonstrated the critical roles that digital technologies like cloud computing (Carolan 2022), machine learning (Lei 2024), and the internet of things (Yadav et al. 2021b) play in maximizing the traceability, operational effectiveness, and risk management of food supply chains and agriculture as discussed in Table 7.
4. Researching the economics of cross‐border agricultural food supply chains: (Barrett et al. 2022) proposed that the agricultural food value chain attracts a substantial amount of direct foreign investment. Using methods of lifespan assessments to track environmental policy actions in agricultural products was covered by (Gava et al. 2019). Social dimension: supporting research on international networks of agri‐food production and making recommendations motivated by corporate social responsibility (Wang et al. 2024). Environmental dimension: the focus is on assessing how particular product supply chains, such as those for milk, apple dessert, and regional agriculture and food supply networks, integrate environmental policies into their operations (Grote et al. 2021). Dimensions of security and resilience: the research mostly focuses on security‐influencing elements, security assurance studies, and an evaluation of the agricultural food supply chain's resilience (Wang et al. 2023).

**TABLE 7:** Future research directions for AI, blockchain, and IoT‐enabled sustainable agri‐food supply chains.

Future direction	Proposed research direction
Integrated AI–Blockchain–IoT frameworks	Develop unified platforms connecting sensing, secure data management, and intelligent decision‐making
Explainable AI (XAI)	Improve transparency and stakeholder trust in AI‐driven agricultural decisions
Blockchain interoperability	Develop standardized mechanisms allowing different blockchain and supply‐chain platforms to communicate
Edge AI and edge computing	Enable real‐time processing closer to farms, sensors, warehouses, and transportation systems
Federated learning	Enable collaborative AI model development while minimizing direct sharing of sensitive agricultural data
Green blockchain	Investigate energy‐efficient consensus mechanisms and environmentally sustainable blockchain architectures
Digital twins	Develop virtual representations of farms, logistics networks, warehouses, and food products for simulation and optimization
Smart cold‐chain systems	Combine IoT sensing, AI prediction, and Blockchain traceability to reduce spoilage and food waste
Carbon‐footprint monitoring	Use integrated digital technologies to quantify and reduce emissions throughout the AFSC
Autonomous robotics	Integrate AI and IoT‐enabled robots into harvesting, sorting, inspection, and logistics
Real‐world validation	Conduct longitudinal studies and large‐scale pilot projects rather than relying mainly on conceptual frameworks

## 10 Conclusion

The incorporation of advanced technologies like blockchain, the Internet of Things, and artificial intelligence is driving a revolutionary change in the agri‐food supply chain. These developments have enormous potential to improve supply chain traceability, transparency, and efficiency while tackling important problems including resource optimization, waste minimization, and food safety. IoT devices allow for real‐time monitoring of agricultural and logistical activities, while blockchain guarantees safe and unchangeable data sharing, promoting confidence among stakeholders. Through intelligent automation, decision support, and predictive analytics, AI helps stakeholders make data‐driven decisions. Despite these encouraging developments, the industry still confronts several obstacles. Widespread acceptance is hampered by technical issues such as data interoperability, expensive implementation, standardization, explainability, governance, and cybersecurity threats. Socioeconomic variables, such as resistance to change and the digital divide, also need to be carefully considered to guarantee sustainability and fair access. It will be essential for legislators, business executives, and IT companies to work together as the agri‐food supply chain develops. To fully utilize these technologies and create a resilient, sustainable, and customer‐focused agri‐food ecosystem, the industry must solve these issues and promote innovation.

## Author Contributions

**Zainab Jabbar:** writing – original draft. **Atif Liaqat:** writing – original draft, writing – review and editing. **Samreen Ahsan:** software, methodology. **Rabia Iqbal:** formal analysis, visualization. **Adnan Khaliq:** resources, conceptualization. **Muhammad Farhan Jahangir Chughtai:** writing – review and editing, supervision. **Furukh Faiz:** writing – review and editing, software. **Muhammad Zahid Bashir:** writing – review and editing, visualization. **Tariq Mehmood:** writing – review and editing, validation. **Edouard Bugingo:** writing – review and editing, visualization. **Muhammad Zubair Khalid:** writing – review and editing, validation.

## Funding

The authors have nothing to report.

## Conflicts of Interest

The authors declare no conflicts of interest.

## Data Availability

Data sharing not applicable to this article as no datasets were generated or analysed during the current study.
